# Early Relaxation Dynamics in the Photoswitchable Complex *trans*‐[RuCl(NO)(py)_4_]^2+^

**DOI:** 10.1002/chem.202000507

**Published:** 2020-08-04

**Authors:** Francesco Talotta, Martial Boggio‐Pasqua, Leticia González

**Affiliations:** ^1^ lnstitute of Theoretical Chemistry Faculty of Chemistry University of Vienna Währinger Strasse 17 1090 Vienna Austria; ^2^ Laboratoire de Chimie et Physique Quantiques, UMR 5626, lRSAMC CNRS et Université Toulouse 3 118 route de Narbonne 31062 Toulouse France; ^3^ Present Address: Laboratoire de Chimie Physique UMR 8000 CNRS/University Paris-Sud University Paris-Saclay, 91403 Orsay, and lnstitut de Sciences Moleculaires d'Orsay, UMR 8214 CNRS/University Paris-Sud, University Paris-Saclay 91403 Orsay France; ^4^ Vienna Research Platform on Accelerating Photoreaction Discovery University of Vienna Währinger Strasse 17 1090 Vienna Austria

**Keywords:** density functional calculations, nitrosyl ligands, photoswitches, relaxation dynamics, ruthenium

## Abstract

The design of photoswitchable transition metal complexes with tailored properties is one of the most important challenges in chemistry. Studies explaining the underlying mechanisms are, however, scarce. Herein, the early relaxation dynamics towards NO photoisomerization in *trans*‐[RuCl(NO)(py)_4_]^2+^ is elucidated by means of non‐adiabatic dynamics, which provided time‐resolved information and branching ratios. Three deactivation mechanisms (I, II, III) in the ratio 3:2:4 were identified. Pathways I and III involve ultrafast intersystem crossing and internal conversion, whereas pathway II involves only internal conversion.

## Graphical abstract

**SHARC attack**: The early relaxation dynamics towards NO photoisomerization in *trans*‐[RuCl(NO)(py)_4_]^2+^ was elucidated by means of SHARC non‐adiabatic dynamics, which provided time‐resolved information and branching ratios. Three deactivation mechanisms (I, II, III) in ratio 3:2:4 were identified. Pathways I and III involve ultrafast intersystem crossing and internal conversion, whereas pathway II involves only internal conversion.

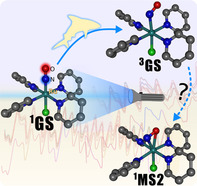

## Introduction

**Scheme 1:**
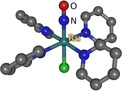
Schematic representation of *trans*‐[RuCl(NO)(py)_4_]^2+^ (py=pyridine).

The photochromism of ruthenium nitrosyl complexes and its capability to photorelease nitric oxide has numerous applications, from material engineering to digital information storage up to the field of photodynamic therapy.^1^, ^2^, ^3^ In this category, *trans*‐[RuCl(NO)(py)_4_]^2+^ (Scheme 1) has attracted considerable attention due to its reversible high photoswitching ability by using different laser wavelengths:^4^, ^5^, ^6^ On continuous light irradiation at approximately 473 nm for 1 h, a conversion yield of approximately 100 % is achieved on a single crystal, while subsequent irradiation at 980 nm regenerates the original crystal.^5^ In contrast, NO dissociation only occurs with low quantum yield in the liquid phase.^7^ Insight into the N→O linkage photoisomerization is thus of utmost importance to design novel electronic devices. However, the only mechanistic information available is based on punctual stationary calculations of selected potential‐energy surfaces (PESs) of such complexes,^8^, ^9^, ^10^, ^11^ and dynamical studies have never been performed for these complexes.

Herein, we report the first study on the relaxation dynamics of *trans*‐[RuCl(NO)(py)_4_]^2+^ to investigate the early steps towards photoisomerization, including both non‐adiabatic internal conversion (IC) and intersystem crossing (ISC) processes. For simplicity, we carried out non‐adiabatic excited‐state dynamics only in the gas phase to describe the processes occurring in the crystal structure using trajectory surface‐hopping methods.^12^ We reveal the role of the singlet and triplet excited states in the isomerization mechanism, establish the branching ratio between different isomerization pathways and identify the most important quenching funnels that make the NO photoisomerization process less efficient.

**Figure 1:**
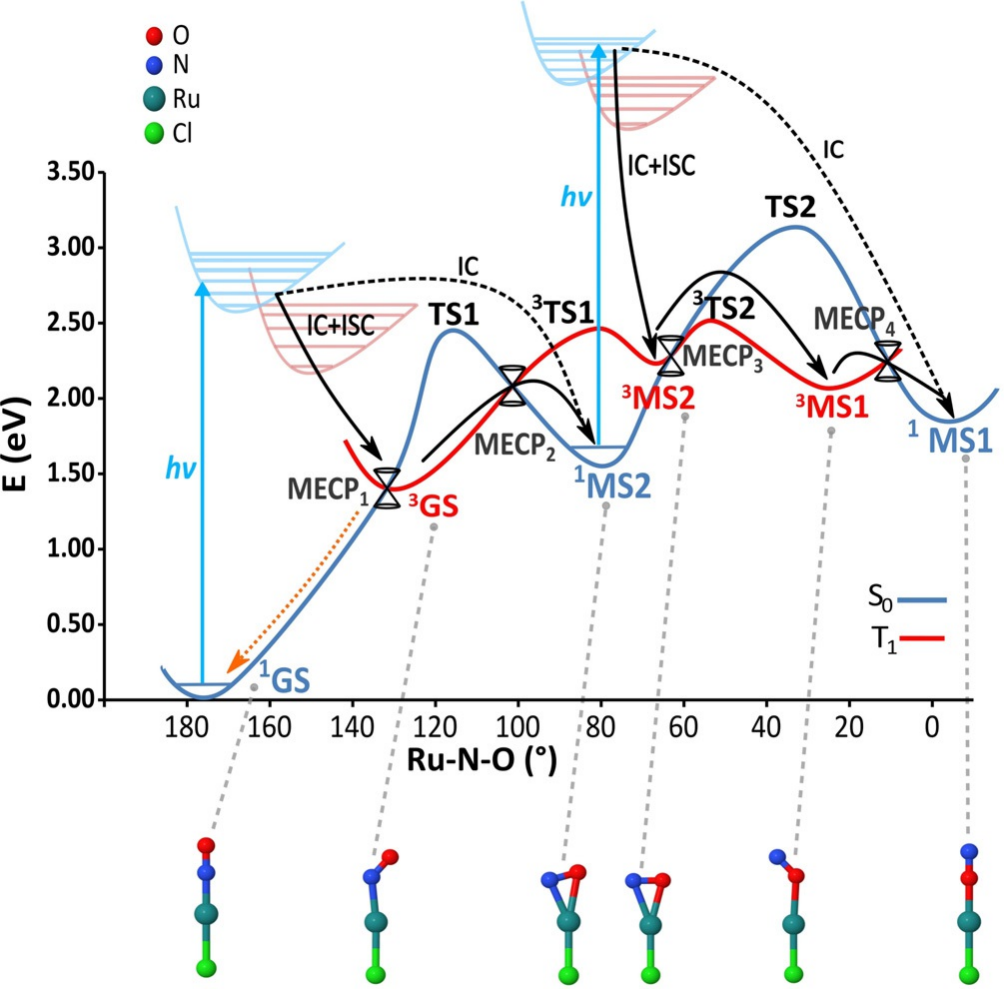
Potential‐energy profiles for the singlet (blue) and triplet (red) electronic states according to the stationary B3LYP calculations of ref. 8. The reaction coordinate is the Ru‐N‐O angle. The three singlet or triplet minima ^1,3^GS, ^1,3^MS2 and ^1,3^MS1 correspond to the N‐bonded, NO‐bonded and O‐bonded isomers, connected by transition states (TS1, TS2 and ^3^TS1, ^3^TS2). The geometry of the Cl‐Ru‐N‐O molecular fragment is shown for each isomer (pyridine ligands have been omitted for clarity). Double‐cone pictograms represent the minimum‐energy crossing points (MECP) between the singlet and the triplet states. Vertical blue lines represent the absorption of two photons from the GS and MS2 isomers. The solid black arrows indicate the photoisomerization pathways I from the GS isomer to ^1^MS2 and from ^1^MS2 to ^1^MS1, respectively, involving IC and ISC. The black dashed lines indicate the additional photoisomerization pathways II proposed according to MS‐CASPT2 calculations,^11^ which involve only IC.

To facilitate the interpretation of the dynamical simulations it is useful to review here briefly the results of the available stationary calculations. Previous DFT^8^ and MS‐CASPT2^11^ stationary calculations, complemented by visible absorption spectroscopy,^13^ indicated that nitrosyl photoisomerization is a two‐step reaction with sequential two‐photon absorption and non‐adiabatic transitions. Assuming efficient nonradiative decays by IC and ISC from the singlet excited states towards the lowest triplet state, the DFT stationary calculations of the lowest PESs suggest the mechanism shown in Figure 1. The most stable ground‐state isomer, labelled ^1^GS, is an orange crystal that corresponds to an N‐bonded structure with a Ru‐N‐O bond angle of 180°. Upon absorption of the first blue photon, the complex is excited to a metal‐to‐ligand charge‐transfer (MLCT) singlet degenerate (S_1_ and S_2_) state, from which relaxation following two different nonradiative pathways is plausible. In the first pathway (pathway I, solid arrows in Figure 1), the complex is expected to undergo several IC and ISC processes until it reaches the lowest triplet state T_1_. According to the MS‐CASPT2 calculations,^11^ the substantial spin–orbit couplings (SOCs) between the singlets S_1_, S_2_ and the triplets T_1_, T_2_, T_3_ (SOC values range from ca. 150 to ca. 250 cm^−1^), together with the close proximity of these excited states, should favour the ISC towards the triplets. Once in the T_1_ state, the complex relaxes to the N‐bonded triplet minimum, labelled ^3^GS. From here, the system can either relax back to the ^1^GS isomer through ISC via the easily accessible MECP_1_ (orange dotted line), or proceed towards the metastable ^1^MS2^6^ isomer by another ISC through MECP_2_. According to the DFT energy profiles of Figure 1, the latter route requires surmounting a barrier of 0.67 eV; however, as MS‐CASPT2 shifts the triplet PES upwards by about 0.6 eV,^11^ the barrier between the N‐bonded ^3^GS isomer and MECP_2_ is considerably lower (<0.1 eV), and this suggests an efficient route to the ^1^MS2 intermediate.

The second non‐radiative relaxation pathway (pathway II, black dashed arrow) involves exclusively IC among singlet states. This path leads directly to the NO‐bonded singlet intermediate state ^1^MS2 through a conical intersection between S_1_ and S_0_ near the transition state ^1^TS1. Both time‐dependent DFT (TD‐DFT) and MS‐CASPT2 calculations^8^, ^11^ suggest that the metastable isomer ^1^MS2 can absorb a second blue photon and be re‐excited to a ^1^MLCT singlet state, which again relaxes by following two distinct non‐radiative pathways. In the first, the system undergoes ISC and IC to reach the NO‐bonded ^3^MS2 isomer, from which the ^3^MS1 is accessed after overcoming the barrier associated with ^3^TS2. From ^3^MS1 an additional ISC through MECP_4_ leads finally to the O‐bonded ^1^MS1 isomer (solid black arrows). As in the case of the first photon, the MS‐CASPT2 calculations predict that the last isomer ^1^MS1 can also be alternatively reached by a pathway involving only singlet states (second dashed line in Figure 1). The final photoproduct (^1^MS1) does not absorb in the blue spectral range and it is observed as a stable green crystal.^8^, ^11^, ^13^ In the forthcoming dynamical study, we thus focus on the first part of the isomerization, that is, from ^1^GS to ^1^MS2.

## Computational Details

Ideally, the most appropriate way to describe this complex is with multiconfigurational multistate complete active space second order perturbation theory (MS‐CASPT2).^11^ Unfortunately, on‐the‐fly surface hopping trajectories at the CASPT2 level of theory for a complex of this size are currently out of reach due to the immense computational effort required. An alternative formalism to perform dynamical calculations would be to use precalculated parameterized potential‐energy surfaces (PESs) on which to propagate wave packets, but this would require to know which are the essential coordinates involved in the photoisomerization and hope that these are either very few and then can be computed with a high‐level of theory, such as CASPT2, or that the isomerization can be described, for example, by simple vibronic coupling models.^14^

To avoid a bias of the presumably complex configurational space, in this work we resorted to full‐dimensional trajectory surface‐hopping methods^15^, ^16^ using an affordable level of theory for the on‐the‐fly calculations of required electronic properties. Specifically, we employed the SHARC approach,^16^, ^17^, ^18^ which is able to describe IC and ISC on the same footing, as demonstrated in a photophysical study on the related [Ru(bpy)_3_]^2+^ complex.^19^ TD‐DFT was chosen as best compromise between efficiency and usability. The limitations and weaknesses of this approach are discussed below. In particular, DFT cannot handle potential fragmentation, and the proximity of the S_1_ and S_0_ brings instabilities, which only a multiconfigurational method can properly account for. However, TD‐DFT is the most accurate method that can be computationally used for the system and we expected it to bring new insights complementary to stationary calculations.

Accordingly, the electronic energies, gradients and spin–orbit couplings (SOCs) were obtained on‐the‐fly by using TD‐DFT, for which a new version of the ADF program package^20^ had to be optimized to deal efficiently with the SHARC workflow. Non‐adiabatic couplings were obtained by using wave function overlaps.^21^ As density functional, BP86^22^, ^23^ was chosen, together with the Tamm–Dancoff approximation (TDA).^24^ The choice of this functional is based on the realization that pure functionals, such as BP86, best describe the singlet–triplet gaps of Ru complexes.^25^ Hybrid functionals such as B3LYP deliver better excitation energies; however, for surface‐hopping small errors in state crossings are preferable over small errors in excitations energies that only lead to a shift in the absorption spectrum. Moreover, the character and ordering of the states at the equilibrium geometry predicted by BP86 agree with MS‐CASPT2 taken as a reference.^25^ As the state crossing energetics were much better with BP86 than with B3LYP, the former functional was selected for dynamics. We are nevertheless mindful of some differences between the PESs obtained with BP86 and MS‐CASPT2 (Figure S1 in the Supporting Information), which are a prerequisite to correctly interpret the dynamical results. Further computational details can be found in the Supporting Information.

According to the available experimental data, at the equilibrium geometry only the bright states S_1_/S_2_ are populated by a blue photon.^13^, ^26^ Initially, these two states must be equally populated, as they form a Jahn–Teller degeneracy (see also Figure S1 in the Supporting Information). Thus, trajectories were prepared in the S_1_/S_2_ pair of states and propagated within the lowest three singlet states (S_0_, S_1_, S_2_) and the three lowest triplet states (T_1_, T_2_, T_3_).

## Results and Discussion

### Absorption spectrum and initial conditions

**Figure 2:**
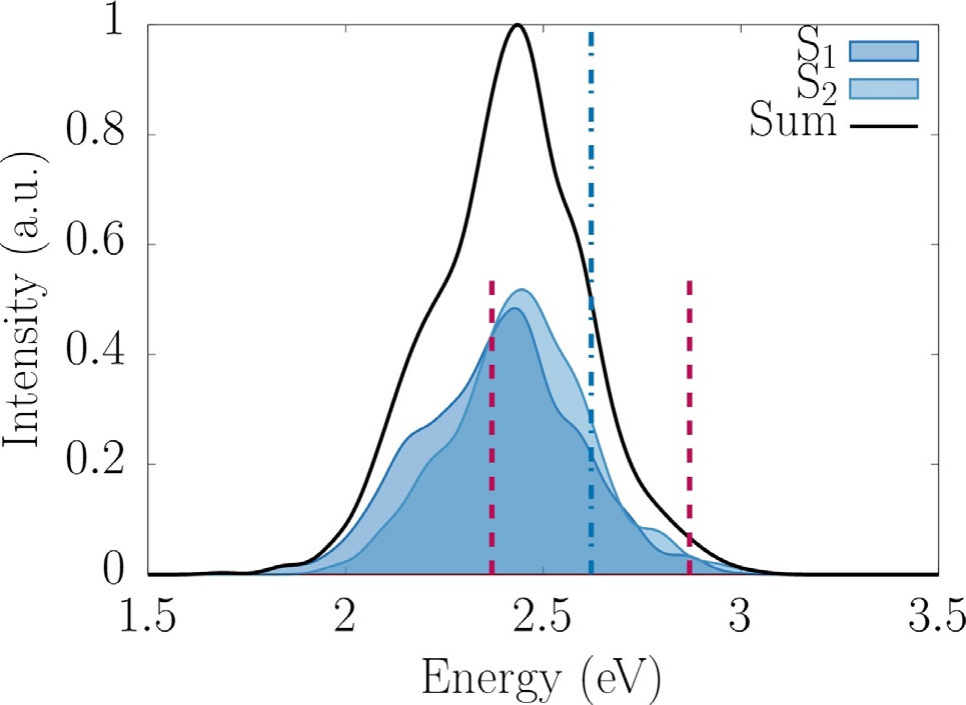
TD‐BP86 convoluted absorption spectra of the *trans*‐[RuCl(NO)(py)_4_]^2+^ molecule from the first two excited states S_1_ and S_2_, obtained from a Wigner distribution of ^1^GS including 500 initial geometries. The vertical blue dashed line represents the experimental excitation energy of 2.61 eV (473 nm) and the red dashed lines delimit the energy window of 0.25 eV centred at 2.61 eV, from which initial geometries and velocities were taken.

We employed a Wigner distribution from 500 initial geometries to calculate the first band of the absorption spectrum of *trans*‐[RuCl(NO)(py)_4_]^2+^. This is composed of the two degenerate ^1^MLCT states, S_1_ and S_2_ (see Figure 2). Because of the degeneracy, the two absorption bands appear almost identical in terms of energies and oscillator strengths. The overall spectrum peaks around 2.44 eV (508 nm), which is in reasonable agreement with the experimental maximum of 2.75 eV (450 nm) measured in acetonitrile,^26^ taking into account the usual underestimation of GGA functionals.^27^, ^28^

The irradiation wavelength was chosen not necessarily to tune the maximum of the S_0_→S_1_/S_2_ absorption band, but to optimize the photoconversion efficiency of the ^1^GS isomer. Accordingly, initial conditions for dynamics were selected from a window of 473 nm (2.61 eV) with ±0.25 eV, as in the photoconversion experiment.^5^ From the original 500 initial Wigner geometries, a total of 144 initial conditions were selected on the basis of the procedure described in ref. 29, of which 74 were instantaneously excited to the S_1_ state and 70 to the S_2_ state. From them, 47 trajectories (33 %) propagated during 1 ps, while the remaining 97 trajectories (67 %) stopped within the first 200 fs, that is, as soon as they reached the zone near TS1 where S_0_ and S_1_ get close in energy, suffer instabilities and the calculation is terminated. Although this is a severe problem of DFT, the behaviour of the trajectories was systematic and allowed us to clearly identify all these “conflicting” trajectories with a particular mechanism.

### Dynamical studies

The dynamical simulations revealed three distinct relaxation mechanisms, which will be discussed separately. Two sets of trajectories nicely confirm the coexistence of pathways I and II predicted by stationary calculations, while a third pathway (denoted as III) emerged from the simulations.

**Figure 3:**
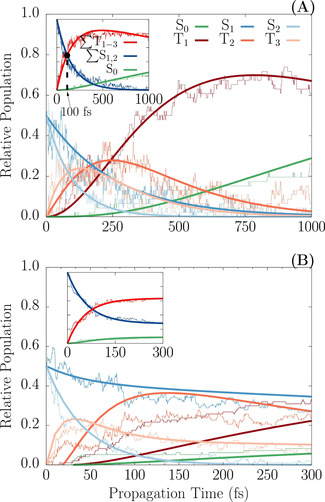
Time evolution of the state populations of the electronic ground and excited states of *trans*‐[RuCl(NO)(py)_4_]^2+^ corresponding to the trajectories that reached 1 ps (A) and 300 fs (B). The inset shows the population of the S_0_ state, the sum of S_1_ and S_2_ populations (∑S_1,2_), and the sum of T_1_, T_2_ and T_3_ population (∑T_1–3_). Thick lines show the fitted functions on top of the corresponding raw population data.

Figure 3 A shows the temporal evolution of the classical population ensemble for the subset of trajectories that reached 1 ps (pathway I). The population data were fitted and bootstrapped^30^ to estimate time constants associated with the various processes. Initially, the S_1_ and S_2_ excited states are equally populated (≈50 %) but within few femtoseconds the triplet states start to become populated due to ISC, so that after approximately 100 fs (see inset of Figure 3 A), the population is inverted from the singlet to the triplet state. The fitting procedure estimates a time constant of 160±30 fs for this process, corresponding to approximately 750 fs to reach a triplet yield of 99 %. This can be considered an ultrafast ISC, albeit slower than those measured and calculated in other transition metal complexes.^19^, ^28^, ^31^, ^32^, ^33^

A closer look at the individual state populations reveals that the moderate decay rate of the singlets is essentially due to slow depopulation of the S_1_ state. Within 300 fs the population of S_2_ drops to 2 %, whereas S_1_ still remains substantially populated (ca. 15 %). A hopping‐event analysis between each pair of states reveals that S_2_ relaxes to the T_3_ state, whereas S_1_ relaxes towards the T_2_ state. Accordingly, the non‐adiabatic transition S_2_→T_3_ is more efficient than S_1_→T_2_. This large difference between the two pairs of states can be explained by the magnitude of the SOC between the excited states. Near the Franck–Condon region, the S_2_/T_3_ pair shows a SOC of approximately 100 cm^−1^, whereas that of S_1_/T_2_ is only approximately 40 cm^−1^. The analysis of the one‐electron transition density matrix of the excited states in the Franck–Condon region shows that the smaller value of the SOC between S_1_ and T_2_ is compatible with the El‐Sayed rule,^34^ in the sense that S_1_ and T_2_ share the same character in their electronic transitions, whereas the wave‐function character of T_3_ is different from that of S_1_ or S_2_ (see Figure S2 of Supporting Information). Upon relaxation to T_3_, the system continues to be deactivated non‐radiatively through IC towards T_2_ with a time constant of 180±20 fs, and eventually to T_1_ with a fitted constant of 150±20 fs. These initial findings underline the role played by the ISC and triplet states in the NO photoisomerization of trans‐[RuCl(NO)(py)_4_]^2+^.

**Figure 4:**
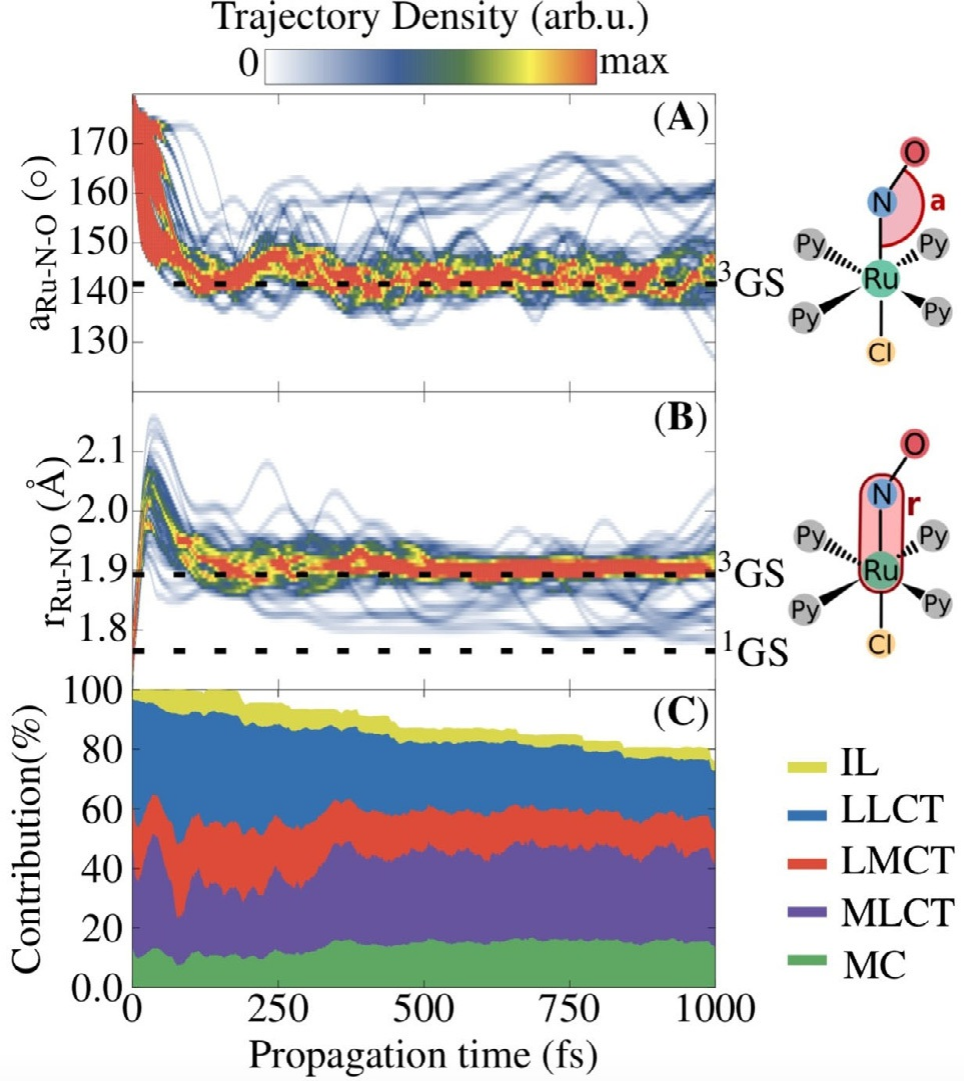
Time‐dependent distribution of the Ru‐N‐O angle (Å) and Ru−NO bond length (B) for the 47 trajectories related to pathway I. The Ru‐N‐O data were smoothed out with a Gaussian smoothing kernel, applied at intervals equal to the Ru‐N‐O vibrational period of 64 fs. Similarly, the Ru‐NO data were smoothed out with a period of 80 fs. The two sets of data were convoluted later, by using a Gaussian convolution kernel. C) Time evolution of the state character (IL, LLCT, LMCT, MLCT and MC) as a stacked population, calculated from the charge‐transfer number decomposition, averaged over all the states of the 47 trajectories.

More insight into the dynamics can be obtained by analyzing the geometrical changes induced by the various relaxation processes discussed above. The time evolution of the Ru‐N‐O angle and Ru−NO distance, which are the most important coordinates related to the N→O linkage isomerization, are shown in Figure 4 A and B, respectively, as a convoluted distribution of the ensemble of trajectories. Additionally, the analysis of the excited‐states character in terms of charge transfer numbers is shown in Figure 4 C as a stacked plot. Depending on the hole and electron directions, charge‐transfer numbers allow^35^ classification of the states as intraligand (IL), ligand‐to‐ligand charge transfer (LLCT), ligand‐to‐metal charge transfer (LMCT), MLCT, or metal centred (MC). The other ligands Py and Cl do not partake in the photoisomerization process, as pointed out in a previous study.^8^

Initially, the geometry of the ^1^GS isomer undergoes coherent bending of the nitrosyl ligand, from almost a linear Ru‐N‐O configuration to approximately 142° in about 160 fs. The analysis of the excited‐states character in Figure 4 C reveals a correlation between the Ru‐N‐O bending and the increase of the MLCT character, at the expense of a decrease of the LLCT character (see the noticeable peak of the MLCT character within the first 80 fs). Such an increase of the MLCT has already been found in stationary calculations of other nitrosyl complexes.^28^, ^36^, ^37^ The angle of 142° reached during this time is consistent with the optimized Ru‐N‐O bending angle of the ^3^GS isomer, de facto the nearest reachable minimum along the N→O isomerization pathway (see Table S7 and Figure S7 in the Supporting Information). Concomitant to the Ru‐N‐O bending, the Ru−NO distance elongates, increasing from approximately 1.76 A (the value of the ^1^GS isomer) to approximately 2.20 Å during the first 60 fs. This elongation is thus also related to the increasing MLCT character of the excited states. Within 160 fs the Ru−NO bond stabilized at approximately 1.90 Å, consistent with the bond length found for ^3^GS. Accordingly, from a structural point of view, the trajectories reach the minimum ^3^GS within 160 fs. However, such a short time is not enough for all the trajectories to relax to the lowest triplet state T_1_, as the S_1_ state is still substantially populated at this stage, also due to the weak SOC between S_1_ with its nearest triplet state T_2_. Furthermore, inspection of the TD‐DFT single‐point calculations showed that the geometry of ^3^GS is also a minimum in the S_1_ PES (see Figure S7 in the Supporting Information). Thus, the discussed Ru‐N‐O angle and Ru−NO bond length also correspond to some trajectories remaining in S_1_.

Upon relaxation to ^3^GS, most trajectories fluctuate in energy until the end of the propagation time, as shown by the thickening of the ensemble in Figures 4 A and B around the ^3^GS equilibrium value. A few other trajectories deviate from this minimum and relax back to the original ^1^GS geometry through ISC between T_1_ and S_0_. A look at the T_1_→S_0_ hopping geometries (see Figure S3 in the Supporting Information) indicates that the ISC occurs near the easily accessible MECP_1_. With TD‐BP86 this crossing point is located in between the ^3^GS and ^1^MS2 intermediate, 0.17 eV above ^3^GS (see Figure S7 and Table S7 in the Supporting Information). The trace of the T_1_→S_0_ hopping is also apparent from Figure 3, as the population of the S_0_ state recovers starting from 250 fs, while T_1_ starts to become depopulated after 750 fs. The depletion of the norm of the one‐electron transition density matrix (see Figure 4 C) is also a consequence of the ISC from T_1_→S_0_. The analysis of the net hops confirms that the T_1_ depletion is directly related to the occurrence of the crossings near MECP_1_ (Figure S3 in the Supporting Information). This relaxation pathway represents a major quenching funnel that could make the entire photoisomerization process quite inefficient, as it reverts the system back to the original isomer ^1^GS. Given the small number of events (only 8 (5 %) trajectories out of 144) that undergo T_1_→S_0_ ISC within 1 ps, the calculated time constant has a large error (1600±600 fs) and should also be considered only qualitatively.

**Figure 5:**
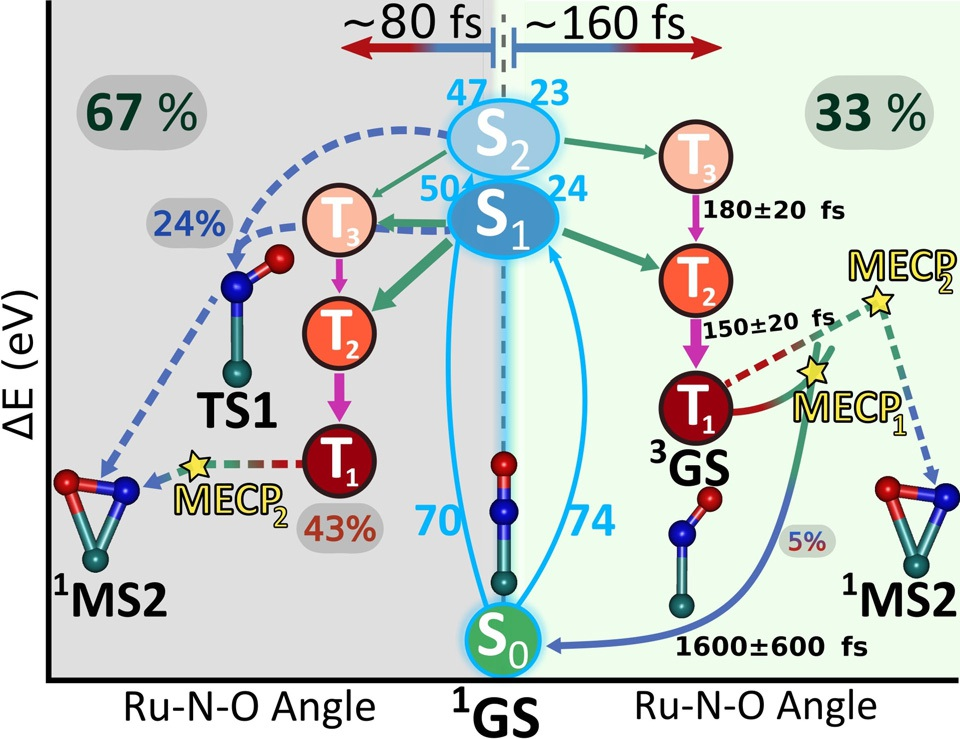
Kinetic and mechanistic model for the photoisomerization of *trans*‐[RuCl(NO)(py)_4_]^2+^, according to the SHARC dynamics. On the right (light green background) the deactivation mechanism related to pathway I and on the left (grey background) that of pathway II. 74 and 70 (light blue digits) trajectories were prepared in the excited states S_1_ and S_2_, respectively. Out of the 74 (70) trajectories, 24 (23) followed pathway I, 21 (14) pathway II, and 29 (33) pathway III. Solid arrows indicate net population transfer between pairs of adiabatic states: green for ISC, blue for IC between singlet states and red for IC between triplet states. The width of the arrows is proportional to the number of net hops (Table S3 and Table S5 in the Supporting Information). Dashed arrows indicate steps not directly observed in the dynamics but extrapolated from stationary MS‐CASPT2 calculations.

The dynamical events related to pathway I are summarized on the right‐hand side of Figure 5. The dynamics simulations could find the mechanism proposed^8^, ^11^ by stationary calculations (Figure 1), although none of the trajectories reach the final intermediate state ^1^MS2 due to the short propagation times and the systematic underestimation of the triplet excitation energies by TD‐BP86 with respect to MS‐CASPT2,^25^ which affects the position of the two T_1_/S_0_ MECPs. In particular, the position of MECP_2_ is crucial, as it allows for the ^3^GS→^1^MS2 passage (see Figure S7 in the Supporting Information). Because, according to TD‐BP86, MECP_2_ is located 0.62 eV above the minimum ^3^GS (see Table S7 in the Supporting Information), a large barrier must be surmounted to reach ^1^MS2. Thus, the ^3^GS→^1^GS pathway through the MECP_1_ is boosted instead, in accordance with the more affordable (0.17 eV) barrier between ^3^GS and MECP_1_. These facts indicate that TD‐BP86 artificially hinders the ^1^GS→^1^MS_2_ isomerization, which otherwise should be favourable, according to MS‐CASPT2 (smaller gap between the singlet and triplet states). This hand‐in‐hand analysis of the dynamical simulations with the quantum chemical calculations is thus critical to reach a comprehensive interpretation of the complex photochemistry of *trans*‐[RuCl(NO)(py)_4_]^2+^.

We now briefly discuss the mechanisms behind the trajectories related to pathways II and III, which account for 64 % of the trajectories. These trajectories are terminated within 30–200 fs, as soon as the S_1_ and S_0_ state are less than approximately 0.2 eV around TS1 and the multireference character of the ground state wave function becomes significant. Figure 3 B shows that within 200 fs the singlet population reaches 35 % and the triplet population 65 %; however, on normalizing the 64 % with respect to the 144 trajectories, these rates correspond to 24 % of singlet and 43 % of triplet population. The associated values of the Ru‐N‐O angle and Ru−NO bond length (see Figure S4 in the Supporting Information) show that all the trajectories halt between ^3^GS and TS1. The singlet trajectories undergo a series of IC transitions bringing population to S_1_ and S_0_ in the region around TS1 (see Figure S4 in the Supporting Information). None of these trajectories stopped on S_2_, and this suggests ultrafast radiationless decay from this state, as was seen in the trajectories pertaining to pathway I. However, in this case depopulation is also due to the rapid energy increase of S_2_ in the region between ^3^GS and TS1 (see Figure S7 in the Supporting Information). Indeed, at ^3^GS the stationary BP86 calculations predict an S_1_–S_2_ energy gap of 0.32 eV, whereas at TS1 this gap becomes 0.7 eV. On the other hand, in the same region the S_0_ energy increases with increasing Ru‐N‐O bending angle, whereas the S_1_ energy decreases (see Figure S7 in the Supporting Information) and, as a result, the S_0_–S_1_ energy gap reduces. Although near the transition state TS1 the gap was calculated to be 0.24 eV by stationary calculations (see Figure S7 in the Supporting Information), the dynamical simulations demonstrate that these two states can get closer, supporting the presence of a S_1_/S_0_ conical intersection near TS1 that can promote efficient formation of ^1^MS2 through the singlet manifold, as suggested by the MS‐CASPT2 calculations.^11^ It can thus be assumed that the S_1_ state will undergo IC through this conical intersection, with the two main ground‐state relaxation pathways leading to ^1^GS and ^1^MS2. Because the momentum associated with the Ru‐N‐O angle is mainly decreasing at the time when the trajectories crash (see Figure S5 in the Supporting Information), we expect the relaxation path to ^1^MS2 to be favoured over the backward return to ^1^GS for inertial reasons (pathway II shown in Figure 1 and dashed blue arrows of Figure 5, left). To support this scenario, 20 crashed trajectories were restarted, half from an Ru‐N‐O angle of 100°, and half from 90°, ready to overtake the S_1_/S_0_ near‐degeneracy region, and indeed ^1^MS2 is readily attained within 50–100 fs (see Figure S6 in the Supporting Information), which confirms this hypothesis. The remaining trajectories that halted on a triplet state underwent efficient ISC and IC transitions (Figure 3 B), similar to pathway I, but here the ISC is faster (80 fs). It appears that these trajectories also reach the TS1 region, as observed in the S_1_ relaxation along pathway II, and again this causes SCF convergence failure. According to TD‐BP86 stationary calculations, the T_1_ energy in this TS1 region is similar to that of T_1_ at MECP_2_ (see Figure S7 in the Supporting Information). Reaching this crossing point involves no energy barrier and little structural change. Pathway III is thus a hypothesis that relies on the assumption that the T_1_ population can be transferred to S_0_ by ISC via the accessible MECP_2_.

## Conclusion

We propose that NO photoisomerization in *trans*‐[RuCl(NO)(py)_4_]^2+^ can proceed by three mechanisms coexisting with a ratio of about 3:2:4 during the first 200 fs. Crucial to each pathway is the presence of non‐radiative ISC and IC, which compete on pathways I and III, whereas IC alone is present on pathway II. Pathway I is followed by 33 % of the trajectories, which undergo IC and ISC towards the triplet minimum ^3^GS in the T_1_ PES. ISC occurs with a time constant of 160±30 fs. Within the 1 ps simulation time, a small amount (5 %) of trajectories come back to the starting isomer ^1^GS, and this highlights one of the possible quenching funnels that can slow down the entire photoisomerization process. 24 % of the trajectories belong to pathway II and halt in the region near TS1 in singlet state S_1_ or S_0_ within the first 200 fs. This mechanism does not involve ISC, but only IC relaxation towards TS1 or the nearby conical intersection to eventually reach ^1^MS2 by another IC. Finally, 43 % of the trajectories halted on a triplet state during the first 200 fs in the region near TS1 (pathway III), which similarly to pathway I, involves ISC and IC, but has a faster ISC process with a time constant of 80 fs. Once in the triplet state these trajectories could either reach ^1^MS2 by ISC through the nearby MECP_2_ or undergo barrierless relaxation back to the minimum ^3^GS.

The three mechanisms highlight the versatility of *trans*‐[RuCl(NO)(py)_4_]^2+^ as a photoswitching agent, and evidence the complexity of ruthenium nitrosyl photochemistry. The present study also illustrates the complementarity of stationary quantum chemical calculations and dynamical simulations, as high‐level quantum chemical calculations are indispensable to assess the validity of the different regions of the PES and critically interpret the outcome of the dynamics.

## Conflict of interest

The authors declare no conflict of interest.

## Supporting information

As a service to our authors and readers, this journal provides supporting information supplied by the authors. Such materials are peer reviewed and may be re‐organized for online delivery, but are not copy‐edited or typeset. Technical support issues arising from supporting information (other than missing files) should be addressed to the authors.

Supplementary
