# Supplementary material for: Early Relaxation Dynamics in the Photoswitchable Complex trans‐[RuCl(NO)(py)4]2+
Source: Chemistry. 2020 Aug 4;26(50):11522–8. doi: 10.1002/chem.202000507 (PMC7539916; doi:10.1002/chem.202000507)
Supplement: Supplementary file 1 — Supplementary [file CHEM-26-11522-s001.pdf]

Author Contributions

L.G. Conceptualization: Equal; Funding acquisition: Equal; Resources: Lead; Supervision: Equal; Writing - Review & Editing: Equal
M.B. Conceptualization: Equal; Funding acquisition: Equal; Supervision: Equal; Writing - Review & Editing: Equal
F.T. Data curation: Lead; Formal analysis: Lead; Investigation: Lead; Software: Lead; Writing - Original Draft: Lead.
